# Maternity protection entitlements for non-standard workers in low-and-middle-income countries and potential implications for breastfeeding practices: a scoping review of research since 2000

**DOI:** 10.1186/s13006-023-00542-8

**Published:** 2023-01-30

**Authors:** Catherine Pereira-Kotze, Alison Feeley, Tanya Doherty, Mieke Faber

**Affiliations:** 1 School of Public Health, University of the Western Cape (UWC), Cape Town, South Africa; 2 South African Medical Research Council (SAMRC) Developmental Pathways for Health Research Unit (DPHRU), School of Clinical Medicine, University of the Witwatersrand, Johannesburg, South Africa; 3 Health Systems Research Unit, South African Medical Research Council (SAMRC), Cape Town, South Africa; 4 Non-communicable Diseases Research Unit, South African Medical Research Council (SAMRC), Cape Town, South Africa; 5 Department of Dietetics and Nutrition, University of the Western Cape, Cape Town, South Africa

**Keywords:** Maternity protection, Non-standard work, Breastfeeding, Low-and-middle-income countries

## Abstract

**Background:**

Recommended breastfeeding practices contribute to improved health of infants, young children, and mothers. Access to comprehensive maternity protection would enable working women to breastfeed for longer. Women working in positions of non-standard employment are particularly vulnerable to not accessing maternity protection entitlements. The objective of this scoping review was to determine the current research conducted on maternity protection available and accessible to non-standard workers in low-and-middle-income countries and any potential implications for breastfeeding practices.

**Methods:**

Nine databases were searched using search terms related to maternity protection, non-standard employment, and breastfeeding. Documents in English published between January 2000 and May 2021 were included. The approach recommended by the Joanna Briggs Institute was used to select sources, extract, and present data. The types of participants included in the research were female non-standard workers of child-bearing age. The core concept examined by the scoping review was the availability and access to comprehensive maternity protection entitlements of pregnant and breastfeeding women. Research from low-and-middle-income countries was included. The types of evidence sources were limited to primary research.

**Results:**

Seventeen articles were included for data extraction mainly from research conducted in Africa and Asia. Research on maternity protection for non-standard workers mostly focused on childcare. Components of maternity protection are inconsistently available and often inaccessible to women working in non-standard employment. Inaccessibility of maternity protection was described to disrupt breastfeeding both directly and indirectly, but certain characteristics of non-standard work were found to be supportive of breastfeeding.

**Conclusions:**

Published information on maternity protection for non-standard workers is limited. However, the available information indicates that non-standard workers have inadequate and inconsistent access to maternity protection rights. The expansion of comprehensive maternity protection to all women working in positions of non-standard employment could encourage significant social and economic benefits.

## Background

Maternity protection refers to labour rights that can contribute to promoting health and well-being of children and their mothers. The International Labour Organisation (ILO) describes comprehensive maternity protection as a set of entitlements that should be made available to working women when they are pregnant or following childbirth, including: a period of maternity leave; cash and medical benefits while on maternity leave; health protection at the workplace; employment protection (job security) and non-discrimination; at least one daily breastfeeding break and, where possible, childcare facilities [1]. If working women who had recently had a baby were to receive comprehensive maternity protection, this would contribute to creating an environment that protects, promotes and supports more women to breastfeed for longer [2].

Near universal breastfeeding in children under five could prevent 823 000 child deaths and up to 98 243 deaths among women from breast cancer, ovarian cancer and diabetes annually [3, 4]. A Cost of Not Breastfeeding Tool has estimated global economic losses due to not breastfeeding to be USD 341.3 billion or 0.70% of global gross income [4]. Achieving recommended breastfeeding rates has a role in contributing to the achievement of the Sustainable Development Goals [4, 5] and confers many health, economic and development benefits to infants, young children, mothers and society in general [6]. Optimal infant and young child feeding practices result in short- and long-term improvements to infant and child health and development that continue throughout the lifecycle including reduced health care costs; health, economic and emotional benefits for the mother; and environmental sustainability [3, 7, 8]. Despite evidence and guidance, rates of exclusive breastfeeding (EBF) for the first six months remain low globally and do not meet established targets in most countries [8, 9]. Many women struggle to continue breastfeeding upon return to work due to lack of support [8, 10].

Current maternity protection legislation and guidance in most countries focuses on women employed in permanent, full-time positions. Furthermore, research on maternity protection mainly focuses on maternity leave and cash payments during maternity leave while excluding other components of comprehensive maternity protection (health protection, job security, non-discrimination, breastfeeding breaks, and childcare). Chai, et al. (2018) reviewed the maternity leave and cash payment components of maternity protection in 38 low-and-middle-income countries (LMIC), and found an increase in early initiation of breastfeeding, EBF and continued breastfeeding with extended legislated paid maternity leave [11]. Maternity leave is also associated with longer breastfeeding duration together with other health benefits including lower infant mortality, improved immunisation rates, decreased morbidity and reduced maternal postpartum depression [12, 13, –15].

Globally, informal employment is growing but informal work is not adequately acknowledged in research and policy [16]. Informal employment refers to a large and heterogeneous group of working arrangements covering enterprises and employment relationships that are not legally regulated or socially protected [17]. Workers can have informal jobs in formal or informal sectors. We have chosen to use the term non-standard employment as a broad term encompassing various categories of employment relationships, including temporary employment, part-time and on-call work, multi-party employment, disguised or dependent self-employment as well as informal work arrangements in the formal sector [18]. However, the various words used to refer to non-standard employment were included in the search strategy as described in the methods. Common examples of non-standard workers include domestic workers, farm workers, people in contract positions and any workers employed by agencies. Child caring priorities such as breastfeeding compete with activities to generate income. Women working in positions of non-standard employment are particularly vulnerable to not accessing maternity protection. Globally, over 60% of employed people work informally, and in LMIC this proportion is higher; in Africa as much as 86% of employment is informal [19]. However, the various terms used in the literature mean that it is challenging to accurately measure the workers represented in each of these categories.

Most parents are not able to access paid parental leave, breastfeeding breaks, and childcare support [18]. Paid maternity leave and breastfeeding support in the workplace have direct benefits to infants and young children, mothers, employers, and businesses [20], including improved productivity in the workplace and decreased absenteeism [21]. Proximity of the mother and infant enables breastfeeding. There is currently a gap in policy alignment between health recommendations to exclusively breastfeed until six months and the International Labour Organisation Maternity Protection Convention guidance for 14 weeks of maternity leave. Furthermore, most country legislation on maternity protection is insufficient, not comprehensively available and not adequately implemented [20]. There is acknowledgement and there are recommendations, both globally and nationally, that research on the implementation of comprehensive maternity protection is urgently needed, especially for women working in the ‘informal’ sector [8, 22].

The objective of this scoping review was to determine the current research conducted on maternity protection available and accessible to non-standard workers in LMIC and any potential implications for breastfeeding practices.

## Methods

We undertook a scoping review of the literature. A scoping review was used since there is limited literature available on maternity protection for non-standard workers and scoping reviews are appropriate to describe a topic still being defined and researched, and that may be complex and heterogeneous in nature [23]. A preliminary search for existing scoping and systematic reviews conducted on 9 July 2020 revealed no existing reviews on this topic. A protocol developed and reviewed by all authors guided the process followed and was not registered but is available on request. The methods for this scoping review follow the Joanna Briggs Institute (JBI)recommendations [24], based on the Preferred Reporting Items for Systematic Reviews and Meta-Analyses (PRISMA) extension for scoping reviews [24]. The scoping review used the following stages: determining the research question; identifying relevant studies; selecting studies (screening); data extraction (charting the data); and data summary and synthesis of the results. The scoping review questions were: What components of maternity protection (such as health and economic benefits) are available and accessible to non-standard workers in low-and-middle-income countries? What are the potential implications of accessing maternity protection on breastfeeding practices of non-standard workers in low-and-middle-income countries?

### Eligibility criteria

Research was included if it related to the availability of and accessibility to comprehensive maternity protection of pregnant and breastfeeding women working in positions of non-standard employment in LMIC. Evidence sources were limited to primary research. Only documents in English were included, due to the time and resources that would have been required for translations from other languages to English. Any literature published in the last 20 years (since the ILO’s Maternity Protection Convention was finalised in 2000) was included. It is acknowledged that males have a role to play in supporting women to access components of maternity protection and that they have a role in supporting (or sometimes hindering) women to breastfeed [25]. This scoping review focused on research involving women, since they are the rights holders with regard to maternity protection and there is still much improvement required for women directly, before investigating the complexity of gender norms and addressing the supportive role of partners, fathers, husbands, and males.

### Information sources and search strategy (study selection)

A three-step search strategy was used:

1. During August 2020, a preliminary search of the following nine databases was conducted using various combinations of search terms: JBI Database of Systematic Reviews and Implementation Reports, Cochrane Database of Systematic Reviews, EBSCOhost, JSTOR, PubMed, SA ePublications (Sabinet), SAGE Journals Online, ScienceDirect and SpringerLink. The words contained in the titles and abstracts of documents obtained were analysed to determine the appropriate search terms to use.
2. All nine databases were then systematically searched during August 2020 (with an updated search conducted in May 2021) using the final list of search terms decided upon:
  [“maternity protection” OR “maternity benefit” OR “maternity leave” OR “paid maternity leave” OR “health benefit” OR “health protection” OR “medical benefit” OR “medical protection” OR “social benefit” OR “social protection” OR “economic benefit” OR “economic protection” OR “job security” OR “job retention” OR “non-discrimination” OR “breastfeeding break” OR “lactation program” OR “childcare”] AND [(Non-standard OR informal OR temporary OR contract OR agency OR part-time) AND (employee OR employment OR work OR sector)] AND [breastfeeding].
  The various search engines required different Boolean algorithms and the search terms were adapted to cater for these requirements. These search terms were developed from the main research questions and identified by piloting various combinations and strings of keywords in PubMed to determine search terms that produced documents most relevant to the review question. The Senior Librarian at the Faculty of Community and Health Sciences Library reviewed and provided input on the search terms and databases used. The database search included documents from all countries (low, middle, and high-income) and then eligibility based on LMIC was determined at the title and abstract (level 1) screening.

3. The reference lists of identified documents were searched for additional sources. No authors were contacted for additional information as this was not needed.

### Selection of evidence sources

The search results from all nine databases were exported into EndNote X9 referencing software [26] to allow for the identification and removal of duplicate entries. Data (downloaded documents) were transferred to Microsoft Excel for source selection (screening). Two reviewers (CPK and AF) independently screened the first hundred titles and abstracts using the eligibility criteria based on the information in the titles and abstracts (level 1 screening). Thereafter, to ensure reliability, CPK and AF compared the decision-making progress together and reached inter-rater agreement regarding how decisions would be made at level 1 screening. There are conflicting recommendations about whether to include conference abstracts when conducting systematic reviews [27]. We made the decision to remove all conference abstracts since abstracts do not contain comprehensive information and for this review, quite specific details were required (that were not always present in the abstract) to determine eligibility for inclusion. The two reviewers then independently reviewed 1717 documents at the abstract and title level. Results of level 1 screening were compared, discrepancies discussed, and consensus reached regarding the decisions. The two reviewers then screened the 255 documents for which the decision was ‘Yes’ or ‘Maybe’ at the full text level (level 2 screening) according to the eligibility criteria. Where agreement or consensus could not be reached, documents were shared with TD to assist in decision-making. The screening process was iterative and done in several rounds to enable decision making refinements. Justifications were made for all decisions and several meetings took place between the two reviewers to rescreen the evidence to ensure accuracy.

### Charting of the data

A Microsoft Excel spreadsheet with the following headings was developed to extract data from all included articles: citation details (author, year, title, journal name, issue, etc.), study setting, study population, sample characteristics, objectives, study design and methods, key findings related to maternity protection entitlements received and breastfeeding practices, and recommendations. The tool was piloted using three (18%) of the articles and amended where necessary. Charting of the data was done by CPK and reviewed by AF. Methodological quality of included documents was not rigorously appraised, consistent with guidance regarding how to conduct a scoping review [23].

### Collating, synthesising, and reporting the results

All included studies were read, re-read, and summarised by CPK who then used inductive content analysis to code and classify information according to different categories (relevant to the different components of maternity protection and breastfeeding) and themes of similar information from across the studies was grouped. Studies were heterogenous, fairly small and difficult to compare. A first draft of the results was developed by CPK, and this was reviewed by all co-authors.

## Results

### Literature search and identification of included studies

**Fig. 1:**
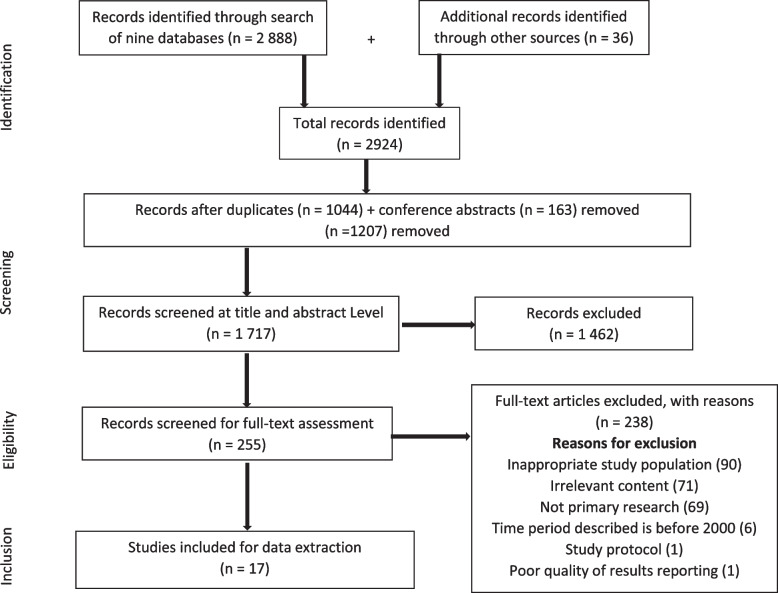
PRISMA flow diagram of the document identification process

A total of 2 924 records were identified. From this, 1 044 duplicates and 163 conference abstracts were identified and removed, resulting in 1 717 unique documents. When screening titles and abstracts (level 1 screening), 255 documents were identified as ‘Yes’ or ‘Maybe’, and 1 462 documents were excluded for not meeting the eligibility criteria. Full-text (level 2) screening was done for the 255 documents, and from this, 17 articles were finally included for data extraction (Fig. 1).

### Study characteristics

**Fig. 2:**
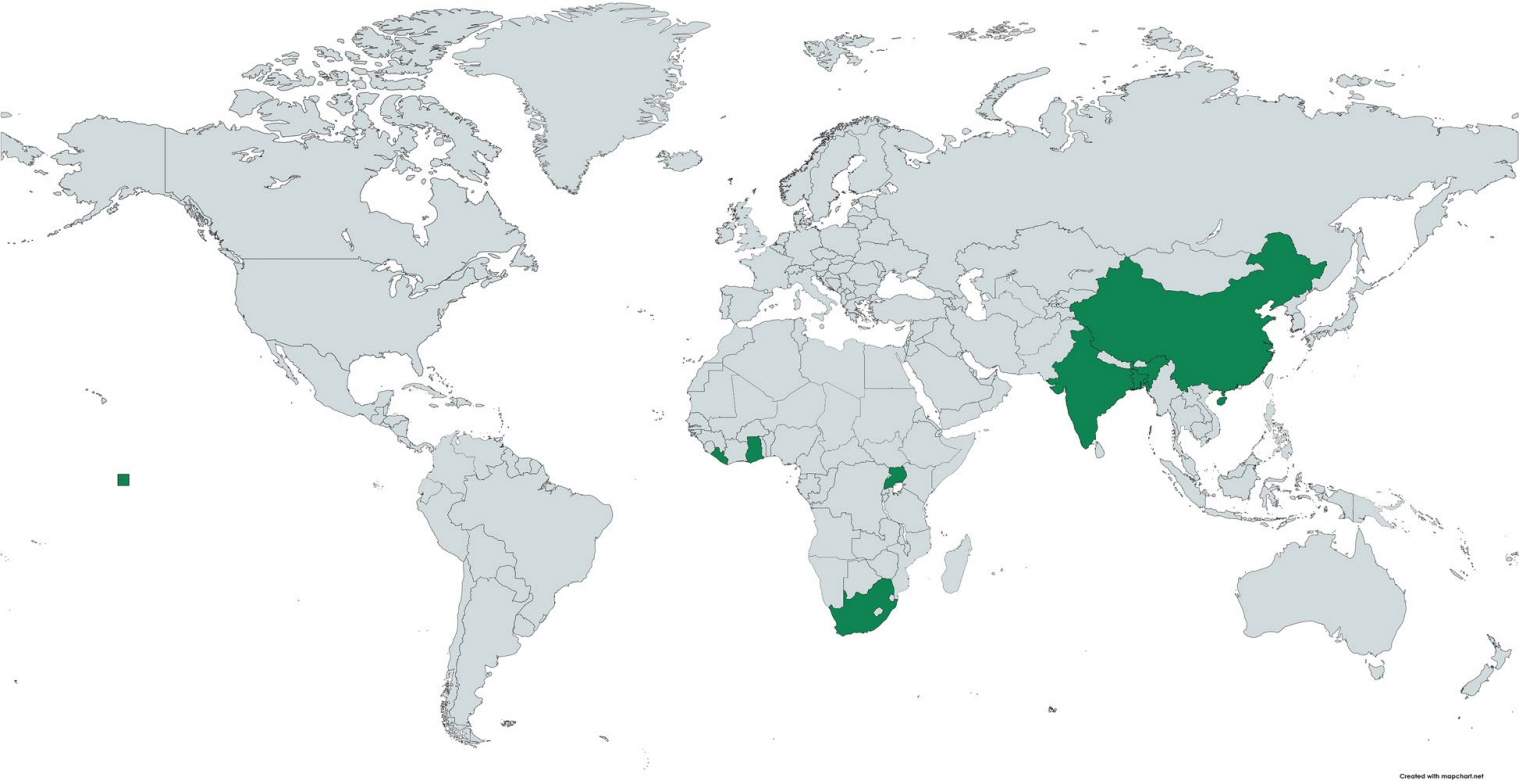
Map indicating geographic locations of included studies

Data was extracted from 17 studies. Four studies included multiple countries (ranging from two to 84 countries) and the 13 studies conducted in single countries were all in either Asia or Africa (four studies in India, three in South Africa, two in Ghana and one each in Bangladesh, China, Liberia and Uganda) (Fig. 2). The types of methods used in the included studies were qualitative including case studies, in-depth interviews and focus group discussions (*n* = 6), mixed methods (*n* = 5), quantitative predominantly using questionnaires or surveys (*n* = 5) and one review. All studies included some reference to how component/s of maternity protection related to breastfeeding practices. Childcare was the most common component of maternity protection that was considered or reported on (*n* = 7), with few studies that considered breastfeeding breaks (*n* = 2), cash payments while on maternity leave (*n* = 2) and one each on maternity leave and health protection. Four studies considered multiple components of maternity and/or social protection more broadly (Table 1). Various types of non-standard workers were described but the term ‘informal’ was used most often to describe women in non-standard employment – either informal worker or women working in the informal economy or informally employed.

**Table 1:** Study characteristics (*N* = 17)

Author, year of publication	Country of origin	Study design	Component/s of maternity protection	Category of non-standard worker
Betancourt, et al. 2013. [28]	India	Qualitative case study (used IDIs & FGDs)	Breastfeeding breaks	Construction workers, including migrant workers
Brahic & Jacobs. 2013. [29]	Ethiopia, Tanzania, Uganda	Action research, mixed methods – structured quantitative questionnaire & IDIs	Health protection at work Maternity leave Job security Breastfeeding breaks	Horticultural farm workers
Nair, et al. 2014. [30]	India	Qualitative: FGDs	Cash benefits Childcare	Women enrolled in the Mahatma Gandhi National Rural Employment Guarantee Act (MGNREGA) – agricultural workers
Alfers. 2016. [31]	Brazil, Ghana, India, South Africa, Thailand	Cross-sectional, qualitative (FGDs)	Childcare	Women informal workers – sampled from associations for waste pickers, street traders (informal vendors), and home-based workers which dominated the sample, but also included domestic workers and agricultural workers
Diji, et al. 2017. [32]	Ghana	Descriptive, cross-sectional: structured, quantitative questionnaire	Maternity leave	Self-employed
Kabir & Maitrot. 2017. [33]	Bangladesh	Qualitative – IDIs and FGDs	Childcare	Women living in ‘slums’ (mostly low-paying, informal jobs)
Ghosh & Kochar. 2018. [34]	India	‘Differences in differences’ approach. Questionnaire and anthropometry (weight, height)	Income support to rural pregnant women—Indira Gandhi Matritya Sahayog Yojana (IGMSY)	Women employed in home production or in the informal economy
Stumbitz, et al. 2018. [35]	Ghana	Qualitative exploratory case study: document review; IDIs	Comprehensive maternity protection	Informal economy defined enterprises that are small, unregistered private unincorporated businesses that do not provide written employment contracts
Chen, et al. 2019. [36]	China	Cross-sectional, mixed methods – quant questionnaire & qual semi-structure interviews	Breastfeeding breaks Maternity leave Childcare	Formal vs. informal in different occupational fields (agriculture, industry, business and white collar)
Gupta, et al. 2019. [37]	84 countries, 69 /84 (82%) LMIC	WBTi assessment of 10 indicators at country level	Comprehensive maternity protection	Women working in the informal or unorganised sector
Horwood, et al. 2019 [38]	South Africa*	Descriptive and cross-sectional, quantitative survey	Childcare	Informal traders and domestic workers
Horwood, et al. 2020. [39]	India and South Africa*	Qualitative – FGDs	Breastfeeding breaks, childcare	Domestic workers, market traders and home-based workers
Kumeh, et al. 2020. [40]	Liberia	Mixed methods: Sequential explanatory, case–control design – quant survey and qual IDIs	Social protection (income support) and maternity support	Mothers attending school or vocational training, informal work or piecemeal work, subsistence agriculture
Luthuli, et al. 2020 [41]	South Africa*	Longitudinal mixed-methods study – quantitative questionnaire and IDIs at 3 time points	Maternity leave Breastfeeding breaks Childcare	Domestic work, home-based work, informal trading, and hairdressing
Nabunya, et al. 2020. [42]	Uganda	Community-based cross-sectional survey: semi-structured (quant) questionnaire	Maternity leave Breastfeeding breaks Childcare	Women working in general shops, food shops/restaurants, furniture shops, small scale salons and markets
Horwood, et al. 2021. [43]	South Africa*	Mixed methods longitudinal cohort study – quant structured questionnaire, IDIs & group photovoice	Childcare	Various including domestic workers (6), hairdressers (5), home-based workers (4), informal vendors (1), fuel attendant (1) and informal tuck shop owner (1)
Kumar. 2021. [44]	India	Review	Health protection	Tobacco cultivation / harvesting (farmers) – ‘casual’ work relationships

*IDI* In-depth interview, FGD Focus group discussion

^*^ The four publications from South Africa reported different sets of results from research conducted under the same umbrella project

### Components of maternity protection addressed by included studies

From the 17 studies included, there were various aspects of comprehensive maternity protection addressed by each study (Table 2). Only two studies addressed comprehensive maternity protection. Childcare (*n* = 9) was the most common component of maternity protection addressed, followed by breastfeeding breaks (*n* = 6) and maternity leave (*n* = 5). Only a few studies considered cash payments of income support (*n* = 3), health protection (*n* = 2) and job security (*n* = 1). None of the included studies considered access to medical benefits or non-discrimination due to pregnancy or breastfeeding.

**Table 2:** Components of maternity protection addressed by included studies (*N* = 17)

Component of maternity protection	Number of studies
Childcare	9
Breastfeeding breaks	6
Maternity leave	5
Cash payments or income support	3
Comprehensive maternity protection	2
Health protection	2
Job security	1
Access to medical benefits	0
Non-discrimination	0

Two main themes and four sub-themes were identified across the 17 studies included and are presented in Table 3. The first theme described is access to maternity protection and from the included studies, this was shown to be inconsistent and that maternity protection was inaccessible, and that this inaccessibility disrupts breastfeeding. The second theme was the relationship between non-standard work and breastfeeding, whereby certain characteristics of non-standard work were described as enabling of breastfeeding while others directly obstruct breastfeeding.

**Table 3:** Themes and sub-themes identified across the included studies

Themes	Sub-themes
Access to maternity protection	Inconsistent and inaccessible maternity protection Inaccessibility to maternity protection disrupts breastfeeding
Relationship between non-standard work and breastfeeding	Characteristics of non-standard work can enable breastfeeding Some aspects of non-standard work can indirectly obstruct breastfeeding

### Inconsistent and inaccessible maternity protection

Inconsistent maternity protection that was difficult to access and inconsistently available to non-standard workers emerged as a strong theme. Several studies described how women lacked access to multiple components of maternity protection, including paid maternity leave and breastfeeding breaks [29, 36, 39, 41, 42, –44]. This resulted in women working for as long as possible prior to having giving birth to a child and returning to work before having fully recovered from childbirth because they could not access maternity leave [36, 45]. Mothers in South Africa tried various strategies to cope with unpaid maternity leave, such as using the child support grant received for older children (although insufficient for the additional costs of a new baby), support from the child’s father and/or other family members (cannot be relied on for long), accumulating savings (although, often, non-standard workers earn too little to be able to save) and, where possible, continuing to be paid by the employer during maternity leave [41, 43]. Despite planning to take longer maternity leave, many participants in a small qualitative study in South Africa went back to work earlier than two months after childbirth, with some returning within two weeks due to financial pressures. A small qualitative study in India described absent creche (childcare) facilities even though there was an act recommending all worksites to have creche facilities [30]. Health protection is not always available to non-standard workers. A study from east African horticultural farms described that many pregnant farmworkers had no personal protective equipment to guard against chemical hazards, and this was associated with miscarriages. One report described a woman going into labour on a farm (while at work) with no access to medical care resulting in a stillbirth [29]. In a qualitative study in India, when work sites were far from home, some breastfeeding mothers faced physical problems like pain and swelling of their breasts due to not being able to feed the child for long periods of time [30].

Two studies reported different programmes in India where cash payments were made available to pregnant women and mothers. One programme is a ‘wage-for-employment scheme’ implemented by the government targeting impoverished and food insecure households where one-third of beneficiaries were women [30]. The evaluation of the programme reported that wages were low, and payments often delayed and that any advantages of providing employment and income was outweighed by compromises to childcare and infant feeding. Another concerning finding was that some mothers were coerced to work in the programme through physical violence by family members and then often not allowed to determine how the income was used which was described by mothers as disempowering. The second programme provided income support to rural pregnant women in India and was shown to have implementation challenges due to weak administrative capacity [34]. This resulted in some women receiving income transfers intended during pregnancy after the child’s birth and sometimes after the birth of a second child. However, even then, significant improvements in child weight-for-age Z-scores were reported resulting in improvements in child growth. A possible reason provided for this was that some households borrow against future income and adjust expenditures based on expectations of future cash transfers.

Female non-standard workers often only benefit from informal or discretionary maternity and social protection which can include unpaid leave and flexible working conditions (e.g., bringing the baby to work) [35]. However, this creates inconsistencies for implementation, unequal conditions, and potential exploitation of individuals. A Ghanaian case study described how this has resulted in both supportive practices and discrimination to pregnant women and new mothers coexisting across and within workplaces [35]. While most studies described challenges that non-standard workers experience in accessing maternity protection, one study described one country (Uganda) where some maternity protection entitlements have successfully been extended to the informal sector but the details of what these entitlements were was not described [37].

### Inaccessibility to maternity protection disrupts breastfeeding

The lack of access to certain components of maternity protection by non-standard workers creates direct and indirect barriers to breastfeeding. In a mixed-methods study in China, results from 10 408 breastfeeding mothers showed that informally employed mothers had lower odds of current breastfeeding compared to mothers employed formally [36]. In a Ghanaian study with 240 mothers, almost half of whom were self-employed, short maternity leave was one of the top three breastfeeding challenges [32]. A unique challenge for informal workers related to physical work is that work often does not take place in offices and female informal workers therefore lack access to private, hygienic, safe and/or sufficient space to breastfeed or express milk [27, 45]. It may not be culturally acceptable to breastfeed at work or express in public or at work, especially, for example, for women handling food [45]. Mothers skipping breastfeeds due to working time constraints resulted in early introduction of solids in a Bangladeshi qualitative study [33]. Two studies reported non-standard working mothers spending extended time away from their children, minimising the time available to breastfeed [30, 42]. Informal workers in the agricultural sector were described as working seasonally and during certain seasons (e.g., harvesting), shifts were extremely long. Women working on tobacco farms in India worked 15-h shifts once harvesting started and mothers did not have time to go to their houses to breastfeed [44]. It was also reported as being impractical for some mothers to carry their infants to work [43]. Some mothers reported that a young sibling (aged seven or younger and usually a girl) may carry the baby to the mother’s workplace for breastfeeding [44]. In a qualitative study in Bangladesh, some mothers reported that because they left for work so early, the baby was either sleeping or not hungry [33]. Sometimes workers were allowed breaks, but often there was no time for this [44]. An Indian study also demonstrated that mothers’ employment in a rural employment scheme compromised infant feeding and childcare [30].Therefore, many non-standard workers face a trade-off between work and breastfeeding and mothers’ need to work to earn an income potentially exposes infants to suboptimal feeding practices.

When women returned to work while breastfeeding, they were encouraged to leave expressed breastmilk with the caregiver, but several challenges were described. It is difficult for women to express sufficient milk for the duration of mother and child separation, and some babies found drinking from a bottle challenging. In a study with 18 women in South Africa, only one mother was able to maintain breastfeeding by expressing when she returned to work [41]. Mothers in India and South Africa raised concerns about the safety of expressed breastmilk, describing that it could become spoiled or contaminated [45]. Mothers did not always have a fridge at the workplace, especially in informal settings, to store expressed breastmilk [45]. Some mothers reported that they left expressed breastmilk to be fed to the baby, but it was not always fed to the child in time and sometimes spoiled [33].

### Characteristics of non-standard work can enable breastfeeding

A few studies (*n* = 4) described aspects of informal work that could facilitate breastfeeding. The flexibility of informal work could allow family members to bring the infant to the mother allowing her to breastfeed at work. Some women could ask for longer unpaid maternity leave without risk of losing their job if they can afford this [36]. Research in South Africa reported that the flexibility of informal work means that some mothers can take the infant to work, others can change to working from home and others go between work and home to feed the baby [39, 45]. Certain types of informal work appeared to be adaptable to breastfeeding, for example in a South African study with 247 participants, although informal traders were more likely to be currently breastfeeding than domestic workers, domestic workers felt more comfortable with both taking their baby to work and expressing at work than informal traders [37]. Women in more senior positions may have more autonomy which can enable longer duration of breastfeeding. Among women working informally in Uganda, those who owned the business or worked in managerial positions had higher rates of EBF than women working as cleaners, assistants, waitresses or in sales [42].

### Some aspects of non-standard work can indirectly obstruct breastfeeding

Mothers in non-standard work are often unable to access maternity leave, cash payments while on maternity leave and breastfeeding breaks**.** When these mothers attempt to combine work and breastfeeding, they can experience reduced incomes and/or job insecurity. Examples of this were provided from research in South Africa, where some mothers were unable to compete for work or had fewer clients resulting in lower incomes, and some lost their jobs when they brought their infants to work. Similarly, mothers who chose to work from home with the baby had less time to work, lower productivity or didn’t meet work targets also resulting in lower incomes [39, 41, 43]. Other mothers who took their infants to work reported having to start early, leave late or miss breaks (including potential breastfeeding breaks) to ensure that work was completed [41]. From a study in Liberia, it was described that “the time-intensive search for piecemeal work” caused mothers and infants to be separated for extended time periods, disrupting breastfeeding [40].

Access to good quality childcare that is affordable for parents is limited for non-standard workers who cannot always afford formal childcare and therefore often make use of family members, friends or neighbours to care for their children [30, 44]. Mothers in South Africa and India reported being uncertain about the safety and quality of the childcare available [30, 43]. While flexibility was described as a positive characteristic of non-standard work, it can also be problematic since the unpredictability of non-standard work makes it difficult for mothers to plan or establish consistent childcare arrangements [43]. Vulnerable working mothers in Bangladesh reported that often multiple caregivers were involved in feeding the child and would feed to their own convenience and that some caregivers had limited nutrition and hygiene knowledge [33]. This meant that even when mothers’ nutrition knowledge was improved through an intervention, caregivers looking after infants for most of the day did not have the same knowledge [35]. It was also reported that often when children are left in non-parental care when mothers return to work, breastfeeding is stopped or other foods and/or fluids are introduced while breastfeeding (i.e., mixed feeding), sometimes resulting in the early introduction of solids [40, 41]. A qualitative study in India, conducted with mothers working in the construction industry reported that even when women had access to childcare and two daily breastfeeding breaks, infants were still given supplemental formula [28].

## Discussion

The research conducted on maternity protection for non-standard workers has focused on childcare with little research available on other components of maternity protection. Non-standard work was mostly described in the literature as informal employment and research was mainly conducted in Africa or Asia. The results show that generally, workplaces of mothers in the informal sector are not supportive of breastfeeding. Inaccessibility to maternity protection for non-standard workers was mostly described to disrupt breastfeeding directly and indirectly, while certain characteristics of non-standard work were shown to enable breastfeeding. While two studies reported that non-standard working women sometimes experience a trade-off between work and breastfeeding, not a lot of research has been conducted on all components of comprehensive maternity protection available and accessible to non-standard workers in LMIC and potential implications for breastfeeding.

Previous research in LMIC has shown that formal employment is associated with a lower likelihood or shorter duration of breastfeeding compared to non-formal employment or unemployment [46–48]. However, a Ghanaian study with 240 mothers in this scoping review reported that self-employed mothers were more likely to EBF than unemployed mothers. A possible explanation provided was that unemployed mothers may think their nutritional status is inadequate to meet the infant’s needs from breastmilk [32]. The results of this scoping review together with previous research indicates that there can be both advantages and disadvantages to different types of employment (formal vs. non-formal) and unemployment. Furthermore, unintended negative consequences of maternity leave legislation were reported in Columbia, where women who had children experienced a drop in salaries and were more likely to be unemployed or work informally to cope with having a child [49].

Policy and stakeholder analyses conducted in five South Asian LMIC revealed that maternity protection in those countries excluded informal workers and made clear recommendations for the need to expand maternity protection to include women employed in non-standard arrangements such as in atypical forms of dependent work or informal or unorganised sectors where many women work [50, 51, 52, –54]. However, the 2016 Lancet Breastfeeding Series acknowledged that even if legislation and accountability mechanisms to ensure maternity protection and workplace breastfeeding support were implemented in countries globally, these would not reach women employed in non-standard arrangements (or women involved in vocational training or attending school) [8]. This could be because some social security programs require prior contribution to access entitlements (such as paid maternity leave) [55] and since non-standard workers are often excluded from formal schemes, they may not be registered for nor able to access this prior contribution. Therefore, additional strategies are needed to assist all working mothers who are breastfeeding. A significant shift in social norms is required to normalise support for all working mothers, especially for the many women working informally who make a significant contribution to countries’ economies.

Certain components of maternity protection such as breastfeeding breaks, cash payments while on maternity leave and childcare (reported in the results of this scoping review) have been more researched than others (health protection, medical benefits, non-discrimination, and job security). The ILO and Women in Informal Employment: Globalizing and Organizing (WIEGO) have written recommendations on provision of quality childcare services for informally employed women workers. Global reviews on policies for breastfeeding breaks concluded that labour laws often excluded non-standard workers (self-employed, part-time workers, domestic workers, agricultural/ seasonal workers, family-business workers, or small enterprise workers). Some LMIC (Sri Lanka, Morocco, Dominican Republic, Indonesia, Thailand, South Africa, and India) have extended legislation to include non-standard workers [56] and it has been recommended that certain components of maternity protection, such as providing breastfeeding breaks should be readily feasible to extend to women working in the informal economy [55]. Several costing estimates have recently been conducted in LMIC to illustrate that providing cash payments while on maternity leave (through maternity cash transfers) for women working informally is financially feasible for governments [57, 58, –60]. A systematic review of pregnancy support programmes in LMIC recommended that in a country like South Africa which has comprehensive social security programmes, that extending the current social assistance for children to begin during pregnancy would be feasible and operationally simple if integrated within existing social support programmes [61].

The categorisation of countries as LMIC can be helpful but LMIC represents a very heterogeneous sample, especially in terms of the proportion of informal workers in various LMIC. For example, in Brazil, 46.0% of workers are informal while in Ghana, 90.1% of workers are informal [62]. Therefore, interventions for maternity protection may need to be quite different for countries with such different profiles, even though they are both LMIC.

The links between informal work, social protection and maternal and child health have previously been highlighted as a research gap [16]. Others have recommended that research is needed to understand the interactions between employment and workplace conditions, and health outcomes of pregnant women, mothers and their children [16]. Innovative models of social protection are also required, that are less dependent on employers or workplaces to deliver employment entitlements, and labour regulations should create conditions that empower working mothers to care for themselves and ensure their children reach their health and development potentials [16].

It has been argued that it should be relatively simple and require little infrastructure to increase accessibility to certain provisions of maternity protection for non-standard workers, such as breastfeeding breaks, non-discrimination and job security and allowing time off for antenatal and postnatal check-ups (part of medical benefits or access to healthcare) [38, 55]. For components of maternity protection such as cash payments while on maternity leave and childcare (to ensure close proximity for breastfeeding), employers and governments are going to need to commit to investing in ensuring these are accessible for non-standard workers. The provision of good quality, accessible and public childcare services has previously been recommended as a key policy intervention with potential to improve productivity and incomes of informally working women [31]. Provision of good quality and affordable childcare can improve women’s labour force participation and have economic benefits [63]. In addition to this, childcare close to work could ensure proximity for breastfeeding, a challenge described by many non-standard working mothers. It is important that women’s right to provide the best care to their children is prioritised.

Future research is required to determine the accessibility to all components of comprehensive maternity protection by non-standard workers in LMIC. Since non-standard work arrangements are diverse and can be unpredictable, flexible and heterogeneous approaches are required to ensure that all women can access maternity protection which could in turn provide a workplace environment supportive of breastfeeding [38]. Future reviews could consider grey literature and published original research in languages other than English. The studies included in this research were all conducted in certain regions of Africa and Asia. There appears to be a gap in research on maternity protection for non-standard workers and implications for breastfeeding in LMIC in South America as well as certain regions (e.g., North Africa and South-east Asia). It would also be helpful to have more regular systematic evidence reviews on the topic of maternity protection, non-standard employment and breastfeeding practices. Such evidence is needed to motivate for policy change in the areas of social justice, gender equity and the protection, promotion, and support of breastfeeding.

### Limitations

Since no software was used for the screening (source selection) process, there is the possibility of human error in the exact reporting in the PRISMA diagram. This review was limited by the inclusion of articles published only in English and exclusion of grey literature. The language limitation may have resulted in papers from some LMICs being excluded.

## Conclusions

This scoping review of original research published in English in peer-reviewed journals illustrated that published information on maternity protection for non-standard workers is limited. Available information indicates that non-standard workers have inadequate and inconsistent access to maternity protection which contributes to further marginalisation and inequalities of an already vulnerable group. While some research has been conducted on certain components of maternity protection for non-standard workers (maternity leave, cash payments, breastfeeding breaks, and childcare), hardly any research has been conducted on health protection and medical benefits, non-discrimination, and job security as components of maternity protection for non-standard workers. The expansion of comprehensive maternity protection to all women working in positions of non-standard employment could encourage significant social and economic benefits.

## Data Availability

The data analysed during this scoping review of the literature are publicly available.

## Abbreviations

EBF: Exclusive breastfeeding
ILO: International Labour Organization
JBI: Joanna Briggs Institute
LMIC: Low-and-middle-income countries
PRISMA: Preferred Reporting Items for Systematic Reviews and Meta-Analyses
