# Correction: Maternity protection entitlements for non-standard workers in low-and-middle-income countries and potential implications for breastfeeding practices: a scoping review of research since 2000

**DOI:** 10.1186/s13006-023-00547-3

**Published:** 2023-02-21

**Authors:** Catherine Pereira-Kotze, Alison Feeley, Tanya Doherty, Mieke Faber

**Affiliations:** 1 School of Public Health, University of the Western Cape (UWC), Cape Town, South Africa; 2 South African Medical Research Council (SAMRC) Developmental Pathways for Health Research Unit (DPHRU), School of Clinical Medicine, University of the Witwatersrand, Johannesburg, South Africa; 3 Health Systems Research Unit, South African Medical Research Council (SAMRC), Cape Town, South Africa; 4 Non-communicable Diseases Research Unit, South African Medical Research Council (SAMRC), Cape Town, South Africa; 5 Department of Dietetics and Nutrition, University of the Western Cape, Cape Town, South Africa

**Correction: Int Breastfeed J 18, 9 (2023)**

**https://doi.org/10.1186/s13006-023-00542-8**

Following publication of the original article [1], it came to the authors' attention that the references and citations from reference 25 onwards were incorrectly numbered: (the former) reference 25 had no corresponding citation and so should have been removed, but this was not done and, as a result, the references and citations from 25 onwards were incorrect (they were each too high by 1). The references and citations have since been corrected in the published article.
